# Temporal and spatial analysis of psittacosis in association with poultry farming in the Netherlands, 2000–2015

**DOI:** 10.1186/s12879-017-2608-1

**Published:** 2017-07-26

**Authors:** Lenny Hogerwerf, Manon M. C. Holstege, Elisa Benincà, Frederika Dijkstra, Wim van der Hoek

**Affiliations:** 1 Centre for Infectious Disease Control, National Institute for Public Health and the Environment, Bilthoven, The Netherlands; 2 Currently: GD Animal Health, Deventer, the Netherlands; 3 Department of Bacteriology and Epidemiology, Wageningen Bioveterinary Research, 8200 AB Lelystad, The Netherlands

**Keywords:** Psittacosis, Avian chlamydiosis, Zoonosis, Poultry, Spatial analysis

## Abstract

**Background:**

Human psittacosis is a highly under diagnosed zoonotic disease, commonly linked to psittacine birds. Psittacosis in birds, also known as avian chlamydiosis, is endemic in poultry, but the risk for people living close to poultry farms is unknown. Therefore, our study aimed to explore the temporal and spatial patterns of human psittacosis infections and identify possible associations with poultry farming in the Netherlands.

**Methods:**

We analysed data on 700 human cases of psittacosis notified between 01-01-2000 and 01-09-2015. First, we studied the temporal behaviour of psittacosis notifications by applying wavelet analysis. Then, to identify possible spatial patterns, we applied spatial cluster analysis. Finally, we investigated the possible spatial association between psittacosis notifications and data on the Dutch poultry sector at municipality level using a multivariable model.

**Results:**

We found a large spatial cluster that covered a highly poultry-dense area but additional clusters were found in areas that had a low poultry density. There were marked geographical differences in the awareness of psittacosis and the amount and the type of laboratory diagnostics used for psittacosis, making it difficult to draw conclusions about the correlation between the large cluster and poultry density. The multivariable model showed that the presence of chicken processing plants and slaughter duck farms in a municipality was associated with a higher rate of human psittacosis notifications. The significance of the associations was influenced by the inclusion or exclusion of farm density in the model.

**Conclusions:**

Our temporal and spatial analyses showed weak associations between poultry-related variables and psittacosis notifications. Because of the low number of psittacosis notifications available for analysis, the power of our analysis was relative low. Because of the exploratory nature of this research, the associations found cannot be interpreted as evidence for airborne transmission of psittacosis from poultry to the general population. Further research is needed to determine the prevalence of *C. psittaci* in Dutch poultry. Also, efforts to promote PCR-based testing for *C. psittaci* and genotyping for source tracing are important to reduce the diagnostic deficit, and to provide better estimates of the human psittacosis burden, and the possible role of poultry.

## Background

Zoonoses, infections transmitted from animals to humans, are an emerging world-wide problem [1]. A still relatively neglected zoonosis is human psittacosis. Psittacosis, also named parrot disease and avian chlamydiosis in birds, is caused by the gram negative, obligatory intracellular bacterium *Chlamydia psittaci* (*C. psittaci*) [2]. It is a common infection in parrots and other birds that is mostly asymptomatic but can be fatal for these animals [3–5]. Birds shed the bacterium through various routes, such as faeces, urine, and nasal secretions [6]. Humans might subsequently inhale very fine droplets or dust particles contaminated with * C. psittaci* [6]. Infection in people can be asymptomatic but may also result in systemic illness, including pneumonia [4, 5]. Even though the pneumonia can be severe, with adequate treatment, fatalities are rare [4].

Human psittacosis occurs worldwide [5]. The disease is notifiable in several countries, including the Netherlands, but significant underreporting is likely [6]. Tests for *C. psittaci* are often not included in routine microbiological diagnostics or consist of serological tests that cannot provide conclusive evidence if only one serum sample is available. Notified cases are therefore likely to represent only a small portion of the actual number of infections. In a recent study in the Netherlands 4.8% (7/147) of patients with community-acquired pneumonia were diagnosed with human psittacosis [7]. Altogether there is an unclear estimate of the public health burden and primary sources of human psittacosis.

Human psittacosis outbreaks are often associated with pet (parrot-like) birds and bird gatherings [4, 5]. Still, psittacosis is found in 465 bird-species nowadays [3]. Wild birds, for example, are mentioned as a possible other source of human psittacosis [8, 9]. Endemicity of *C. psittaci* is reported in poultry, including in commercially kept ducks [10–13], chickens [14, 15], and turkeys [14, 16]. In addition, a recently described * C. psittaci* related species named *C. gallinacea* is found to be predominant in chickens with a so far unknown zoonotic potential [17–19]. Research in 10 Belgian turkey farms revealed 94% of the turkeys to be infected with * C. psittaci* [16]. In Belgian chicken farms, 18 out of 19 studied studied farms were infected with *C. psittaci* [15]. Recent research also points out a role of poultry (i.e. ducks, chickens and turkeys) in human psittacosis infections. Employment in poultry processing plants and/or poultry farms is increasingly demonstrated to be related to outbreaks of human psittacosis [5, 12, 14–16, 20]. People dealing with poultry regularly, for example farmers, poultry processing employees and veterinarians have a higher risk for infection with *C. psittaci* [5]. Previous studies in the Netherlands found a higher incidence of pneumonia around poultry farms [21, 22]. In Australia, rural environments were indicated as a risk factor for psittacosis [23]. These are possible indications that poultry farms might also lead to psittacosis in humans by indirect contact (airborne transmission) [24]. This would have substantial implications for current guidelines, source tracing and screening practices. In the present study, we aim to assess whether human psittacosis notifications in the general population are associated with presence of poultry farming and poultry processing plants in time and space.

## Methods

### Study population and data description

Data on 737 human cases of psittacosis notified in the Netherlands between 01-01-2000 and 01-09-2015, were used. These cases are notified by the regional public health services (GGD) in the online notification database Osiris and fulfil the national notification criteria for psittacosis: a positive polymerase chain reaction (PCR) or a combination of matching clinical characteristics and a fourfold titer rise using serology [6, 25]. Via Osiris, information regarding demographical and epidemiological data (including four digit postal code) was obtained. These anonymised data were available as part of routine surveillance and epidemiological studies at the National Institute for Public Health and the Environment, the Netherlands. Information was present on a possible/suspected occupational link and in some cases a likely origin of infection was mentioned. Cases related to known outbreaks were excluded to avoid distortion of the association under study (*N* = 36), as for these cases the origin of infection was already determined and not related to poultry. Other cases where a possible source of infection was mentioned, were taken into account since no conclusive evidence on the origin of infection was present. Therefore, in total, 700 human cases of psittacosis were included in the study.

### Data on poultry sector

**Fig. 1:**
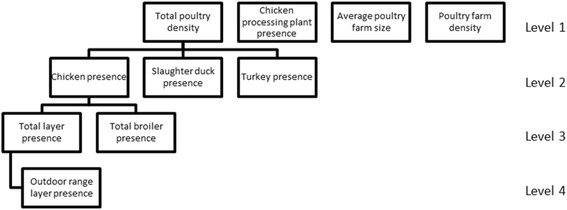
Diagram describing the nested structure of poultry-related variables. The levels grow in specificity, where level 1 is the most general and level 4 is the most specific level

Data on various aspects of the Dutch poultry sector was acquired from the agricultural census of the Netherlands Enterprise Agency (rvo.nl), Ministry of Economic Affairs, which included information about all individual farms with poultry. Aggregated agricultural census data are available from Statistics Netherlands (CBS) [26]. One reference year, 2012, was chosen because of practical issues resulting from municipal reclassifications. In addition, very detailed information about individual farms was only available for the year 2012, including data on exact locations and the number of poultry, divided into different subspecies. Some farms had multiple locations; therefore, in some cases the number of poultry was divided over the locations. When possible, the poultry was re-assigned to one of the locations based on data per location from the internet and a provincial database of mandatory environmental licenses for keeping livestock (province of Utrecht) [27]. Only poultry farms with ≥500 birds in total (all poultry present on the farm combined) were included in the analyses to prevent possible distortion by ‘hobby animals’. Hobby animals were excluded because only information on hobby animals on farms was present, not on hobby animals in people’s backyards because these are not included in the agricultural census. Poultry variables that were used in the study, aggregated at a municipality level, were: poultry density, poultry farm density, average poultry farm size, chicken processing plant presence, chicken presence, turkey presence, slaughter duck presence, layer presence, broiler presence and outdoor range layer presence. The variables are nested in structure and therefore we organized the variables into four levels with a growing specificity (illustrated in Fig. 1). In Table 1, the variables, both on the human and veterinary side, are listed and described briefly.

**Table 1:** Description of outcome- and explanatory variables, at a municipality level

Variables	Description	Unit (per municipality)
Human		
Number of cases notified per total person years	Number of human psittacosis infections notified from 01-01-2000 until 01-09-2015 weighted against the total person years based on the reference year 2012 (population in 2012 times 14 years).	Number of cases / total person years
Animal		
Total poultry density	The density of all poultry combined.	Birds/km^2^
Total poultry farm density	The number of poultry farms per km^2^ times factor 100.	Number of farms*100/km^2^
Average poultry farm size	Average size of a poultry farm.	Birds
Chicken processing plant presence	The presence of a chicken processing plant.	Yes/no
Chicken presence	The presence of chickens (both broilers and layers).	Yes/no
Turkey presence	The presence of turkeys.	Yes/no
Slaughter duck presence	The presence of slaughter ducks.	Yes/no
Total broiler presence	The presence of broilers (including parents of broilers)	Yes/no
Total layer presence	The presence of layers (including parents of layers).	Yes/no
Outdoor range layer presence	The presence of places for layers with outdoor range.	Yes/no

### Wavelet analysis

In order to identify potential temporal patterns in the data of psittacosis notifications we performed wavelet analysis, a technique suited for non-stationary time series as is the case for many epidemiological data [28]. Wavelet analysis uses a local periodic function (the wavelet) to decompose fluctuations of time series observed during a small time interval into a series of different periodicities. The relative importance of periodicities (wavelet power) is then plotted in contour plots as a function of time. In this way, the periodicity and the timing of the fluctuations can both be determined. Further information on wavelet analysis is available elsewhere [29–33].

### Spatial scan statistics

In order to detect spatial clusters of high rates of psittacosis we applied the SaTScan spatial statistics using the SaTScan software (SaTScan version 9.4) [34, 35]. The total numbers of psittacosis notifications (2000–2015) and population numbers per four-digit postal code (reference year 2012) served as the analysis input. The location of the cases was set using the coordinates of the centroid of their four-digit postal codes. A Poisson scan statistic with a significance level of *P* < =0.05 was used and the maximum cluster size was set to 50 km radius, a distance sufficient to detect spatially concentrated clusters [36]. Resulting clusters were visually compared with poultry densities in the Netherlands to see if an association between them was plausible (farm infection status was not known).

### Spatial association between human psittacosis notifications and poultry

We performed Poisson regression analyses in R software (v3.2.3.) [37], with the poultry-related variables as independent variables and the cumulative psittacosis notifications weighted against total person years as the dependent variable. The analyses were performed at the level of the municipality. There were 415 municipalities in the Netherlands in 2012. Neighbouring municipalities are presumably more alike, a phenomenon called spatial autocorrelation [38]. To account for spatial autocorrelation, a random effect term was added to the model. The complexity of the model requires a Bayesian approach, for instance using Markov chain Monte Carlo (MCMC). However, with MCMC, fitting the models would take a long time, therefore we used the much faster Integrated Nested Laplace Approximation (INLA). INLA is specifically suitable for the type of random effect terms we used. For analysis, the poultry-related density variables had to be divided into quantiles. The number of resulting quantiles was set to three or less (present/not present) in case of a lack of variability in density.

Univariable analyses were done for all poultry-related variables, to investigate their possible predictive role in the number of psittacosis cases (weighted against total person years). Subsequently, a multivariable model was created taking into account all variables regardless of the results during univariable analyses. The selection of variables to be included in the final multivariable model was based on the Deviance Information Criterion (DIC), an indicator of the quality of models similar to Akaikes Information Criterion (AIC), commonly used in Bayesian model selection. The nested data structure with four levels (as described under ‘Study population and data description’) required a non-standard approach of model selection. The starting model only included the most specific variable (level four) ‘outdoor range layer presence’. Subsequently, the other variables were added one level at a time. A lower DIC of the new combined model allowed for inclusion of newly added variables. As a general rule, ‘parent’ variables (higher up in Fig. 1) were preferred over the combination of their more specific subset variables, when resulting in similar model quality. Every time a level was added, we looked for the combination of variables that gave the lowest DIC possible. Therefore, it was possible that only a selection of the variables from a specific level were added to the model. To check for robustness of the final model, model selection was also performed the other way round, starting with level one.

## Results

From 01-01-2000 to 01-09-2015, 700 valid human cases of psittacosis were reported. The national median number of notifications per year (2000–2014) was 44 with a range from 12 to 77. The distribution of the psittacosis notifications over four-digit postal codes and municipalities in The Netherlands is summarized in Table 2 and Table 3.

**Table 2:** Number of cases per four-digit postal code

Number of cases per four digit postal code	Frequency
0	3487
1	447
2	91
3	21
4	2

Number of human cases of psittacosis notified per four digit postal code in the Netherlands between 01-01-2000 and 01-09-2015, with exception of cases related to known outbreaks. The median number of cases (*n* = 700 cases) per four-digit postal code area (*n* = 4048 areas) is zero, the mean number of cases is 0.17, the standard deviation is 0.47, and the variance is 0.23

**Table 3:** Number of cases per municipality

Number of cases per municipality	Frequency	Municipality names
0	152	
1	114	
2	60	
3	42	
4	11	
5	12	
6	8	
7	2	
8	2	
9	4	
10	2	Utrecht, Barneveld
11	2	Emmen, Haarlemmermeer
12	2	Arnhem, Enschede
15	1	Haarlem
41	1	The Hague

Number of human cases of psittacosis notified per municipality in the Netherlands between 01-01-2000 and 01-09-2015, with exception of cases related to known outbreaks. For those municipalities with 10 or more cases, the name of the municipality is given. The median number of cases (*n* = 700 cases) per municipality (*n* = 415 municipalities) is 1, the mean number of cases is 1.69, the standard deviation is 2.91, and the variance is 8.46

### Temporal and spatial patterns in the human psittacosis notifications

**Fig. 2:**
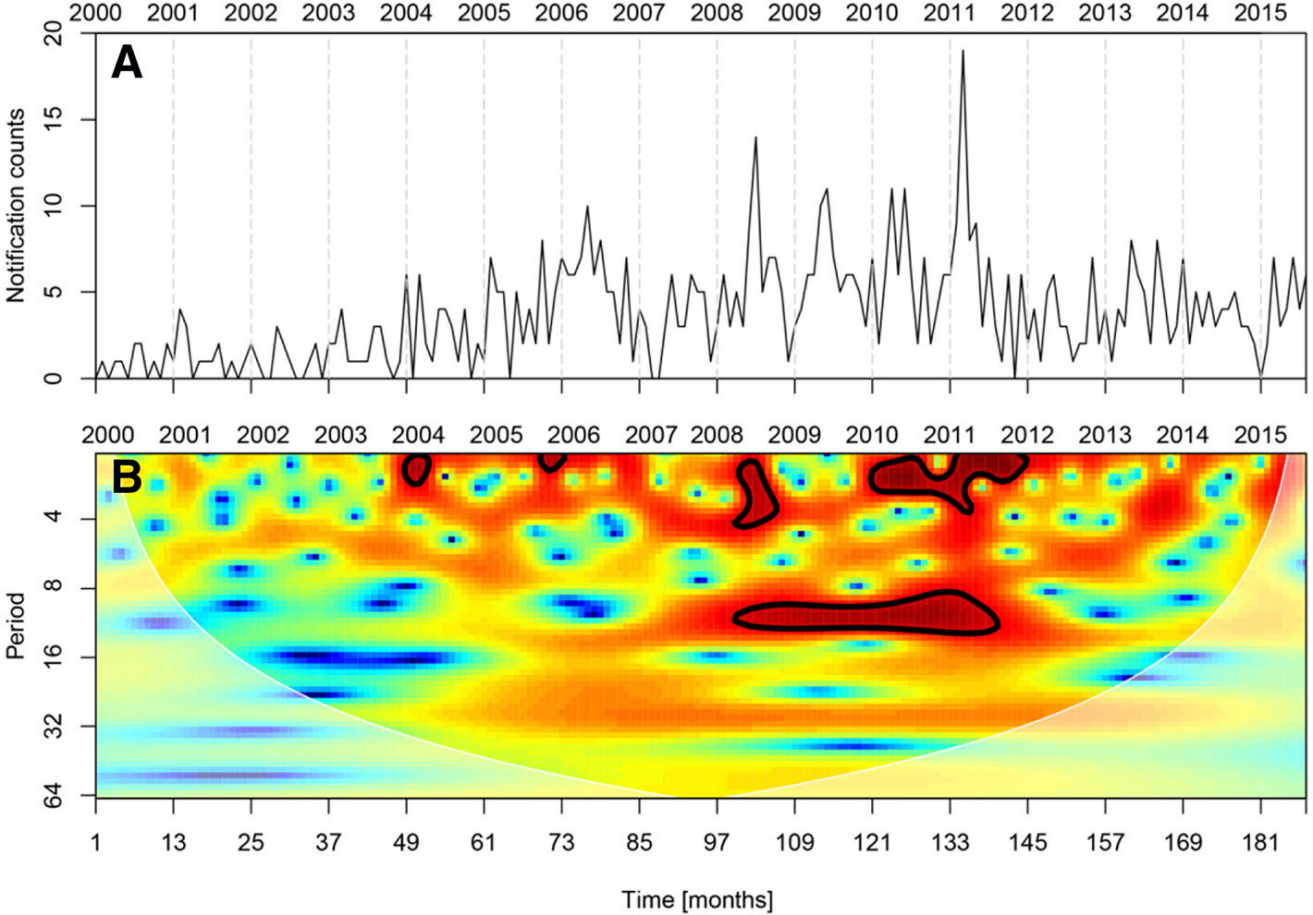
Temporal dynamics of psittacosis notifications from 2000 to 2015 in the Netherlands. **a** Time series of psittacosis notifications plotted on monthly basis. **b** Wavelet power spectra of the psittacosis notifications. Color codes represent wavelet power and areas inside the black contour lines correspond to 95% confidence regions where the power is higher than the power of red noise with the same autocorrelation coefficient as the data. Transparent areas on the left and right hand sides of the plots represent the cone of influence, which is a region where edge effects are important

The temporal dynamics of the psitaccosis notifications show strong variation among the months as well as among the years (Fig. 2a). In the first part of the time series (from 2000 to 2004), the number of notifications is quite low and substantially increases from 2004 onwards. Striking peaks in the number of notifications occur in the period spanning from the beginning of 2008 until the end of 2011. These peaks in notifications always occur during spring-summer months (March–July). This interesting seasonal pattern is confirmed by wavelet analysis, which identified a significant periodicity of about 12 months from the beginning of 2008 until the end of 2011 (Fig. 2b).

**Fig. 3:**
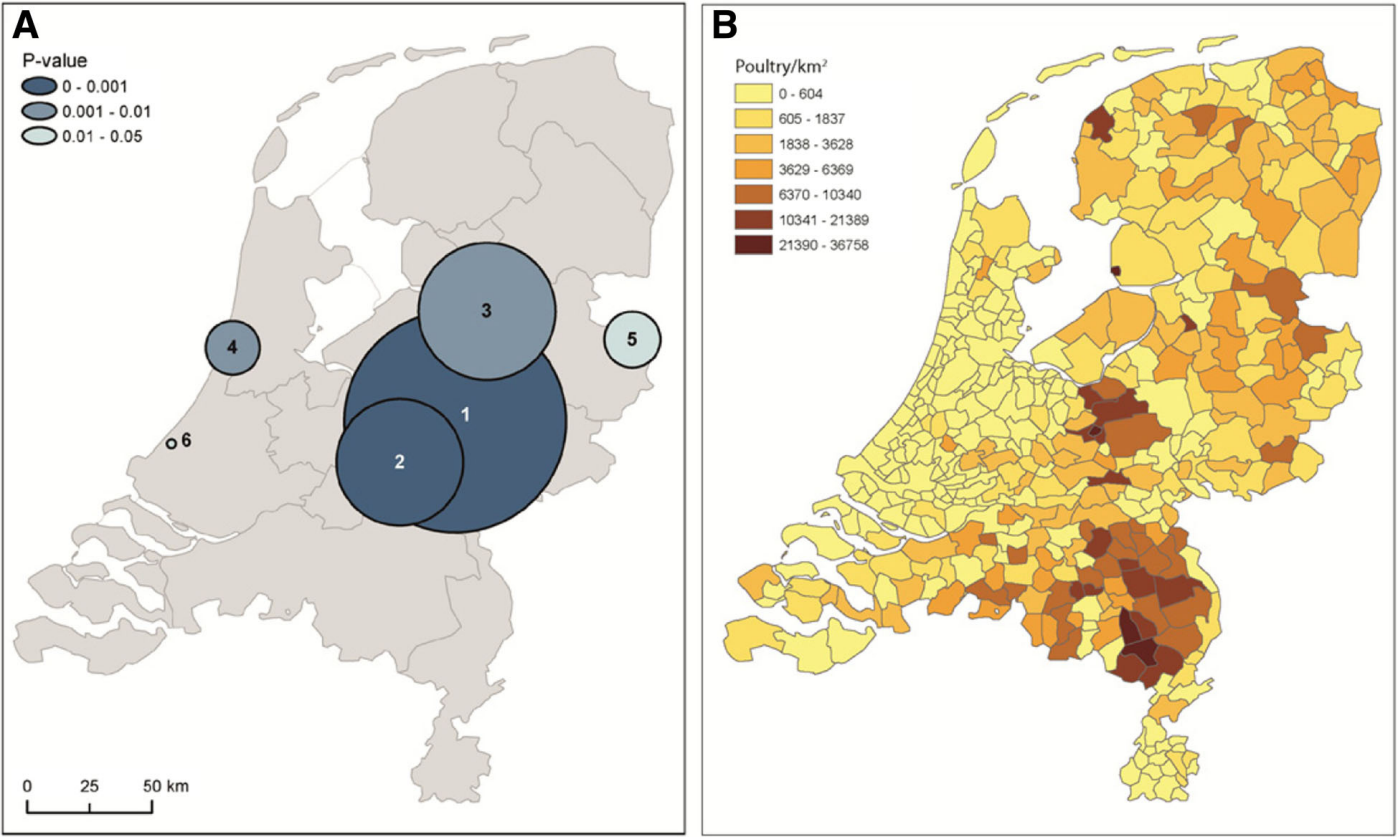
Spatial dynamics of psittacosis notifications in relation to the density of poultry in the Netherlands. **a** Spatial clusters of the total number of psittacosis cases (*N* = 701; cases from known outbreaks were excluded) notified from 01-01-2000 until 01-09-2015. Black circles represent significant clusters (*P* < =0.05) identified whilst imposing a maximum radius of the window of 50 km. **b** Map of poultry density at municipality level. The poultry density data was obtained from Statistics Netherlands (CBS) [26]

Spatial clusters analysis detected six significant clusters (Fig. 3a). F The analysis identified three large spatial clusters (numbers 1/2/3) in the provinces of Gelderland and Overijssel. These include the area of the ‘Gelderse Vallei’, the main poultry production area of the Netherlands and other poultry dense municipalities (Fig 3b). In addition, clusters showed up in areas without poultry, (in Haarlem, number 4 and in and around The Hague, number 6)) or with moderate poultry density (in a rural area near Almelo, number 5).

### Spatial association between human psittacosis notifications and poultry

In univariable analyses, all poultry-related variables with exception of presence of turkeys showed a slightly increased risk for psittacosis (Table 4). However, this increased risk was only statistically significant for chicken processing plant presence. The two multivariable model selection methods (starting at level one and four, selection based on DIC) gave similar results and included slaughter duck presence, chicken processing plant presence and poultry farm density in the final models. However, only chicken processing plant presence and slaughter duck farm presence were significantly associated with a higher incidence rate of psittacosis notifications in the multivariable model (Table 5). For farm density no clear trend was observed. Therefore, the final model is also presented without farm density (Table 5). The incidence rate ratios of the variables in the model altered slightly and were not significant anymore. Therefore, only when correcting for farm density a significant association between slaughter duck presence (Risk Ratio (RR): 1.44 [95% Confidence Interval (CI) 1.01–2.05]), chicken processing plant presence (RR: 1.63 [95% CI:1.03–2.53]) and the human psittacosis notification incidence is visible.

**Table 4:** Univariable RR with 95% CI for psittacosis notifications in the Netherlands, 01-01-2000 until 01-09-2015

Variables	RR (95% CI)
Total poultry density	
0 Birds/km^2^	1.00
0–1150 Birds/km^2^	1.18 (0.91–1.54)
> 1150 Birds/km^2^	1.21 (0.90–1.61)
Poultry farm density	
0 Farms/km^2^	1.00
0–0.05 Farms/km^2^	1.28 (0.99–1.67)
> 0.05 Farms/km^2^	1.06 (0.79–1.42)
Average poultry farm size	
0 Birds	1.00
0–21,000 Birds	1.15 (0.88–1.49)
> 21,000 Birds	1.28 (0.95–1.71)
Chicken processing plant presence	
No chicken processing plant present	1.00
Chicken processing plant present	1.58 (1.01–2.43)
Chicken presence	
No chickens present	1.00
Chickens present	1.20 (0.94–1.53)
Turkey presence	
No turkeys present	1.00
Turkeys present	0.92 (0.59–1.41)
Slaughter duck presence	
No slaughter ducks present	1.00
Slaughter ducks present	1.40 (0.99–1.97)
Broiler presence	
No broilers present	1.00
Broilers present	1.04 (0.82–1.32)
Layer presence	
No layers present	1.00
Layers present	1.06 (0.84–1.32)
Outdoor range layer presence	
No layers with outdoor range present	1.00
Layers with outdoor range present	1.06 (0.84–1.33)

*N* = 415 municipalities

**Table 5:** Final multivariable models for psittacosis infection notifications in the Netherlands, 01-01-2000 until 01-09-2015

Variables	RR (95% CI)	RR (95% CI)
Chicken processing plant presence		
No chicken processing plant present	1.00	1.00
Chicken processing plant present	1.63 (1.03–2.53)	1.55 (0.99–2.38)
Slaughter duck presence		
No slaughter ducks present	1.00	1.00
Slaughter ducks present	1.44 (1.01–2.05)	1.37 (0.97–1.94)
Poultry farm density		*Not included*
0 Farms/km^2^	1.00	
0–0.05 Farms/km^2^	1.26 (0.96–1.63)	
> 0.05 Farms/km^2^	0.94 (0.69–1.28)	

*N* = 415 municipalities

## Discussion

### Temporal and spatial patterns in the human psittacosis notifications

The temporal pattern of psittacosis showed variability in the number of notifications among the months as well as among the years. An interesting seasonal pattern has been identified with peaks in psittacosis notifications during the spring-summer months, but so far an explanation for this pattern is lacking. The number of psittacosis notifications is substantially increased from 2004 onwards. The rise in human psittacosis notifications may be explained by a higher incidence of infection, or more awareness about psittacosis and reduction of underdiagnosis. In the poultry sector, there was a trend towards fewer but larger farms (intensification) and a trend towards new housing systems for animal welfare, with a rise in the available space per bird, including a rise in outdoor range places. Coincidentally, from 2004 onwards, supermarkets in The Netherlands banned eggs from battery cages [39]. Recent studies show that the housing systems for layer hens that replaced the battery cages generate more dust, resulting in higher particulate matter emissions [40].

Spatial cluster analysis identified a large cluster, located in the middle-eastern part of the Netherlands. This cluster included the main Dutch poultry production area: the ‘Gelderse Vallei’ and other poultry dense areas. In the study by Spoorenberg et al. (2016), 7/147 (4.8%) patients with community-acquired pneumonia were diagnosed with psittacosis [7]. Patients were from two hospitals, of which one was located in the poultry dense area of the Netherlands and within the large spatial cluster (cluster one) and the smaller cluster two [7].

While the large cluster could be associated with poultry farming, poultry is very unlikely to play a role in the other two spatial clusters in and around seaside cities. In these clusters, pet birds or wild birds are a possible source of infection [4, 5, 8, 9]. There are likely to be regional differences in the awareness of psittacosis. For instance, the regional public health service and microbiological laboratory in the area of the Haarlem cluster (cluster number 4) were active in research into psittacosis, creating higher awareness [3]. Also, in some regions more diagnostic tests are performed than in other regions with important regional differences in use of PCR or serology as method of choice [7].

### Spatial association between human psittacosis notifications and poultry

Chicken processing plants and slaughter duck farms were significantly associated with human psittacosis notifications. However, the associations were not significant anymore when farm density was left out of the model.

The presence of *C. psittaci* on chicken processing plants and slaughter duck farms and occupational risks for the workers have been reported in studies in Belgium and France [14, 12, 20]. Dickx and Vanrompay (2011) detected *C. psittaci* in air samples of a poultry processing plant and found employees to be infected [14]. Hulin et al. (2015) showed a preference for ducks by * C. psittaci* combined with seroconversion in slaughterhouse employees [17]. Laroucau et al. (2009) showed chlamydial excretion in duck farms and human notifications of psittacosis on those farms with matching PCR patterns [12]. Laroucau et al. (2015) also showed human cases infected with the same *C. psittaci* genotype as the infected chickens they slaughtered [19]. Both chicken processing plants and slaughter duck farms have specific characteristics that may enhance indirect airborne transmission to the general population. In processing plants, stressed chickens enter in partly open crates [41]. Stress triggers the shedding of bacteria and via open crates, particles can easily become airborne [5]. A significant amount of dust is known to be generated at chicken processing plants and *C. psittaci* has been detected inside and at the open entrance of those processing plants [14]. In the case of slaughter duck farms, a lot of dust is created as well, due to the straw that the ducks live on and the relatively low bird density within farms in the Netherlands, allowing for movement and dust creation [42]. Ducks in the Netherlands are accommodated in closed buildings, but particulate matter emerges from ventilation systems and dust release might be enlarged while emptying and re-stocking [40]. In addition to the farm characteristics, it is important to note that ducks can be asympomatically infected while shedding *C. psittaci* heavily [13].

While an association between human psittacosis and chicken processing plants and slaughter duck farms seems possible, the interpretation of the results of the present study remains difficult. Results were not significant anymore when poultry density was removed from the model. In addition, the Dutch national infectious diseases database contains few notifications for psittacosis for which occupational exposure was reported or suspected.

Based on the spatial cluster of cases covering the most poultry dense area of the Netherlands it was expected that poultry farm density would end up in de multivariable model. During model selection, poultry farm density showed to lower the DIC of the model but no significant association with a higher psittacosis incidence was observed, and linked variables like the total poultry intensity did not end up in the final model. Therefore, the role of farm density remains inconclusive.

We did not find an association between psittacosis notifications and outdoor range layer presence. This finding was somewhat unexpected as outdoor range on dry soil (most poultry farms are located on sandy soil) leads to dust formation, and flocks are in close proximity to humans in the surroundings. It has to be noted that outdoor range layer presence is based on the number of places for outdoor range, not the exact number of birds that are outside (part of the time). Therefore, the results should be interpreted with caution.

A limitation of the spatial association analyses is the limited number of municipalities with chicken processing plants in the Netherlands (*N* = 15) and the resulting low power of that analysis. On the other hand, the results gain robustness due to the lack of correlation between chicken processing plants and slaughter duck farm presence mutually and with other variables. Other limitations are that people might have been infected outside their own municipality and that locations of some poultry farms were unclear. In addition, the poultry data originated from 2012 and therefore changes over time in the poultry data could not be taken into account. Also, when a farm had more than one location, we assumed equal distribution over the locations in our analyses. In practice, it is likely that the distribution is not equal and that certain types of poultry are housed at specific locations. Most importantly, we did not know whether farms were infected or not, making it difficult to make statements about a possible association.

## Conclusions

This exploratory research cannot provide firm evidence for airborne transmission of psittacosis from poultry to the general population. The low number of psittacosis notifications available for analysis did not allow for detection of small differences in risk. About 80% of pneumonia patients in the Netherlands are managed in primary care, by general practitioners and current professional guidelines do not include microbiological testing in community-acquired pneumonia (CAP) [43]. Even for the 40,000 to 50,000 patients that are annually admitted to hospital in the Netherlands with pneumonia, more than 85% has an ICD-10 discharge diagnosis of ‘pneumonia, organism not specified’ [44]. An ongoing project aims at promoting PCR-based testing for *C. psittaci* in hospitalized patients with CAP to reduce this diagnostic deficit (http://www.wur.nl/nl/show/Plat4m2Btpsittacose.htm). The project will also determine the presence and prevalence of *C. psittaci* in animal populations, including poultry and the final product should be a ‘one health’ genotyping tool for *C. psittaci*. Together, the project activities are expected to provide better estimates of burden from human psittacosis, and the possible role of different animal reservoirs.

## Abbreviations

AIC: Akaikes Information Criterion
*C. gallinacea*: *Chlamydia gallinacea*
*C. psittaci*: *Chlamydia psittaci*
CAP: Community-acquired pneumonia
CBS: Statistics Netherlands
CI: Confidence Interval
DIC: Deviance Information Criterion
GGD: Community Health Services
INLA: Integrated Nested Laplace Approximation
MCMC: Markov chain Monte Carlo
PCR: Polymerase chain reaction
RR: Risk ratio
